# Relation between task-related activity modulation and cortical inhibitory function in schizophrenia and healthy controls: a TMS–EEG study

**DOI:** 10.1007/s00406-023-01745-0

**Published:** 2024-01-19

**Authors:** Inés Fernández-Linsenbarth, Gema Mijancos-Martínez, Alejandro Bachiller, Pablo Núñez, Víctor Rodríguez-González, Rosa M. Beño-Ruiz-de-la-Sierra, Alejandro Roig-Herrero, Antonio Arjona-Valladares, Jesús Poza, Miguel Ángel Mañanas, Vicente Molina

**Affiliations:** 1 Psychiatry Department, School of Medicine, University of Valladolid, Av. Ramón y Cajal, 7, 47005 Valladolid, Spain; 2 Biomedical Engineering Research Centre (CREB), Department of Automatic Control (ESAII), Polytechnic University of Catalonia, Barcelona, Spain; 3 Institute of Research Sant Joan de Déu, Barcelona, Spain; 4 Coma Science Group, CIGA-Consciousness, University of Liège, Liège, Belgium; 5 Biomedical Engineering Group, University of Valladolid, Valladolid, Spain; 6 Biomaterials and Nanomedicine (BICER-BBN), CIBER of Bioengineering, Madrid, Spain; 7 Imaging Processing Laboratory, University of Valladolid, Valladolid, Spain; 8 Instituto de Investigación en Matemáticas (IMUCA), University of Valladolid, Valladolid, Spain; 9 Psychiatry Service, Clinical Hospital of Valladolid, Valladolid, Spain; 10 Neurosciences Institute of Castilla y Léon (INCYL), University of Salamanca, Salamanca, Spain

**Keywords:** Schizophrenia, Cortical inhibition, Spectral entropy, Modulation, Transcranial magnetic stimulation, Dorsolateral prefrontal cortex

## Abstract

Schizophrenia has been associated with a reduced task-related modulation of cortical activity assessed through electroencephalography (EEG). However, to the best of our knowledge, no study so far has assessed the underpinnings of this decreased EEG modulation in schizophrenia. A possible substrate of these findings could be a decreased inhibitory function, a replicated finding in the field. In this pilot study, our aim was to explore the association between EEG modulation during a cognitive task and the inhibitory system function in vivo in a sample including healthy controls and patients with schizophrenia. We hypothesized that the replicated decreased task-related activity modulation during a cognitive task in schizophrenia would be related to a hypofunction of the inhibitory system. For this purpose, 27 healthy controls and 22 patients with schizophrenia (including 13 first episodes) performed a 3-condition auditory oddball task from which the spectral entropy modulation was calculated. In addition, cortical reactivity—as an index of the inhibitory function—was assessed by the administration of 75 monophasic transcranial magnetic stimulation single pulses over the left dorsolateral prefrontal cortex. Our results replicated the task-related cortical activity modulation deficit in schizophrenia patients. Moreover, schizophrenia patients showed higher cortical reactivity following transcranial magnetic stimulation single pulses over the left dorsolateral prefrontal cortex compared to healthy controls. Cortical reactivity was inversely associated with EEG modulation, supporting the idea that a hypofunction of the inhibitory system could hamper the task-related modulation of EEG activity.

**Supplementary Information:**

The online version contains supplementary material available at 10.1007/s00406-023-01745-0.

## Introduction

In the search for physiological biomarkers of a disease, it has been often fruitful to investigate parameters that are related to its corresponding altered function. Namely, to use the knowledge about the mechanisms underlying the normal function to define biomarkers for the corresponding disease where that function is known to be altered. One of the problems of applying such approach in schizophrenia and other psychiatric disorders is that the substrates of the altered function (i.e., the mental function) are still poorly understood.

Nevertheless, a few facts acknowledged on those substrates may help in the search for biomarkers for schizophrenia or, at least, some of the subtypes that may be included in that syndrome. One of the most important facts in this direction is that complex mental contents, such as those altered in many schizophrenia patients, involve the coordinated and reentrant activity of most cortical regions that takes the form of constantly evolving synaptic assemblies [1, 2], characterized by the synchronous (i.e., phase-locked) firing during a short time. This principle also underlies the neuronal group selection theory proposed by Edelman for higher order consciousness, involving perception, memories, planning, and constitution of self [3]. Similarly, when a mental content reaches the consciousness, the corresponding synchronous firing becomes much more widespread, i.e., the firing extends to the so called “global workspace” [4–6]. Thus, the synthesis of neural assemblies and their permanent evolution seem a good starting point to assess the pathophysiology of, at least, a significant group of patients with schizophrenia.

Considering that a large majority of synapses in the cortex are excitatory, proper neural assemblies evolution, involving their synthesis and cancelation, would only be possible with an adequate inhibitory function, based on gamma-aminobutyric acid (GABA) interneurons [7]. This prevents a global indiscriminate hyperactivation and makes the selection of the adequate synapsis in an assembly possible. The GABA inhibitory system has been consistently reported to be functionally altered in schizophrenia [8, 9] and mood disorders [10]. This suggests that the synthesis of synaptic assemblies underlying mental contents may be hampered in schizophrenia due to an excitatory/inhibitory imbalance. To demonstrate this, it would be necessary to assess in vivo data related to both synaptic assemblies’ synthesis and inhibitory function.

The synthesis of the synaptic assemblies cannot be directly in vivo assessed without invasive techniques, but the electroencephalography (EEG) is a proxy for its evaluation since it reflects the synchronous firing of neuronal groups, which transitorily form a synaptic assembly. Its modulation during a cognitive task corresponds to the rapid formation and cancelation of the assemblies underlying the mental contents related to such task. Thus, EEG, with its high temporal resolution, may be a marker of one of the mental contents’ underpinnings. Among the many metrics that can be derived from EEG activity, Shannon entropy (SE) is a global index of EEG signal irregularity that allows to quantify its changes between different conditions and is, thus, useful as a measurement of task-related modulation [11]. Using this parameter, we have replicated in three different samples that task-related modulation of EEG activity is decreased in schizophrenia [12–14]. This lower modulation was associated with higher pre-stimulus connectivity strength, revealing a basal hypersynchrony [15, 16], and with an increased density of theta spectral power at baseline [17]. These findings are coherent with a decreased inhibitory function as a substrate contributing to that modulation deficit.

Decreased GABA levels have been diversely reported in schizophrenia [18, 19], but these levels might not necessarily reflect the in vivo inhibitory transmission status (*e.g.*, GABA levels may increase for instance in response to a hampered inhibition). In recent years, the combination of transcranial magnetic stimulation (TMS) and EEG has emerged as a powerful tool for assessing both inhibitory and excitatory functions of the cerebral cortex. When a TMS pulse is applied to the cortex, time-locked depolarization of underlying neurons is obtained, and this activity can be recorded by means of EEG electrodes placed on the scalp [20]. In this line, recent assessments of in vivo inhibitory status of the cortex have been published using both single- [21–23] and paired-pulse [24, 25] paradigms combined with EEG. More specifically, previous studies have reported altered GABAergic-mediated neurotransmission in the dorsolateral prefrontal cortex (DLPFC) in schizophrenia patients using TMS–EEG [24–27].

Based on the previous findings stated above, in this study, we aimed to explore the association between the EEG modulation during a cognitive task (as a proxy of the synthesis and dissolution of synaptic assemblies) and the inhibitory system function in the DLPFC in schizophrenia patients using a combination of TMS–EEG. Since said function has been found to be altered in the DLPFC in schizophrenia [8, 9], we chose this region to test our hypothesis, although this dysfunction is unlikely restricted to that region. We hypothesized that the replicated decreased EEG modulation during a cognitive task will be related to a hypofunction of the inhibitory system in the DLPFC in schizophrenia. Since differences in cellular parameters related to inhibitory function, such as GABA_A_ postsynaptic receptors, or GAD 67 enzyme mRNA of GABA transporter are relatively modest in schizophrenia and its values overlap in patients and controls [8, 9], we hypothesized that the association between EEG modulation and the inhibitory system function would differ quantitatively rather than categorically between patients and controls. Patients would show decreased inhibitory function but not a lack of it, and such a decrease would translate into varying magnitudes of EEG modulation deficits. Therefore, in this first analysis, we assessed the relation between EEG and cortical inhibition together in patients and controls.

## Methods

### Participants

Our sample included 27 healthy controls (HC) and 22 patients with schizophrenia, of whom 13 were first episodes (FE). Patients were diagnosed by one of the experienced psychiatrists in the group (VM) according to the criteria of the Diagnostic and Statistical Manual of Mental Disorders 5th edition, considering current mental state, clinical records, and relatives’ information. Exclusion criteria included (a) intelligence quotient under 70; (b) present or past substance dependence (excluding caffeine and nicotine); (c) head trauma with loss of consciousness; (d) neurological or mental diagnosis different to schizophrenia (patients); (e) any current neurological or psychiatric diagnosis (controls); (f) receiving any other treatment affecting central nervous system; and (g) not being safe to undergo TMS. All participants provided informed written consent after full written information before inclusion. The local ethics committee of the Clinical University Hospital of Valladolid endorsed the study (PI 22–263). This work complies with the ethical standards of the Helsinki Declaration of 1975, as revised in 2008.

### Transcranial magnetic stimulation

TMS stimulation was performed using a MagPro X100 stimulator (MagVenture, Denmark) and a figure-of-8 coil. Participants sat comfortably and were instructed to look directly ahead with their eyes open. An EEG cap was fitted to their head and electrodes were placed over the right abductor pollicis brevis (APB) muscle for electromyographic recordings. The resting motor threshold (RMT) was determined over the motor cortical region following the relative frequency method [28], defined as the minimum intensity required to elicit a motor evoked potential (MEP) of > 50 µV peak-to-peak amplitude in at least five of ten subsequent trials. The optimal coil location to determine the RMT was identified as the position that consistently elicited the largest MEPs in the right APB muscle by slightly suprathreshold single-pulse TMS. Afterward, 75 monophasic TMS single pulses at an intensity of 120% RMT were applied over the left DLPFC with randomized jittered inter-stimulus interval from 5 to 7 s to reduce anticipation of the next trial. The coil was positioned in the middle of a line between the F3 and F5 electrodes with a 45º rotation relative to the midline, producing a posterior–anterior current flow in the underlying cortex. This position provides the most accurate estimation of left DLPFC (border of BA9 and BA46) in the absence of neuronavigational equipment [29–31]. To assess potential auditory-evoked potentials that could confound genuine TMS cortical reactivity findings, 40 participants (of them 20 HC) received sham TMS pulses. The sham condition was conducted by placing the coil perpendicular to the left DLPFC.

### Auditory oddball task

During the same session, participants performed a 3-condition auditory oddball task in which 600 stimuli were randomly presented: target (500 Hz tone, probability of 0.2), distractor (1000 Hz tone, probability of 0.2), and standard (2000 Hz tone, probability of 0.6). Each tone lasted 50 ms, with a rise and fall time of 5 ms and an intensity of 90 decibels. The inter-stimulus interval between tones randomly jittered between 1.16 and 1.44 s. Participants were asked to keep their eyes closed and to press the mouse button upon hearing target tones. Target tones were considered attended when followed by a button press. Only attended target tones were considered for further analysis.

### EEG data acquisition

EEG activity was collected using a 64-channel system [Brain Vision (Brain Products GmbH)] following the international 10–10 system. Impedance for all electrodes was lowered to ≤ 5 kΩ. The channels were referenced over Cz during acquisition and re-referenced offline to the averaged activity of all sensors [11, 32]. During the auditory oddball task, the sampling rate was 500 Hz. TMS–EEG data were recorded with a sampling rate of 25 kHz.

### EEG data pre-processing

After recording EEG activity during the auditory oddball task, the following three-step artifact rejection algorithm was applied to minimize electrooculographic and electromyographic contamination [12]: (i) an independent component analysis (ICA) was performed to discard noisy ICA components; (II) the signals were divided after ICA reconstruction into trials of 1 s (from 300 ms prior to the stimulus onset to 700 ms after); and (iii) the trials with amplitudes that exceeded an adaptative statistical-based threshold were automatically rejected [33]. The signals were band-pass filtered between 1 and 70 Hz, and a 50-Hz notch filter was applied to remove the power line artifact.

### Spectral entropy

Spectral entropy modulation was calculated in the auditory oddball task and computed from the normalized continuous wavelet transform (CWT), which is a form of time–frequency representation of a signal that is conceptually related to the short-term Fourier transform [33]. The CWT allows for better detection of dynamic EEG components due to its balance between frequency and time resolution [33]. The time-dependent wavelet-based SE can be defined as follows:

$$SE\left(t\right)=-\frac{1}{{\text{log}}(M)}\cdot {\sum }_{f}WS\left(t,f\right)\dot {\text{log}}\left[WS\left(t,f\right)\right],$$

where *SE* is the spectral entropy (as a function of time) and *WS* is the normalized wavelet scalogram. Specifically, SE was computed in two windows: pre-stimulus (300 ms before stimulus to stimulus onset) and response (150 ms to 450 ms from the stimulus onset, centered around the P300 peak). Afterward, it was averaged in each of the two windows. As in our previous studies, SE modulation was calculated as the difference in SE between response and pre-stimulus windows (Gomez-Pilar et al., 2018b), providing a measure of the degree of the change of signal irregularity across time. Since a decrease in SE in the response window has been robustly observed in healthy controls, normal SE modulation is expected to be expressed in negative values [12, 13, 16]. Complete details of spectral entropy calculation are found in the Supplementary material.

### TMS–EEG signal pre-processing

TMS–EEG signal pre-processing was performed using Fieldtrip [34] and MATLAB (R2021b; The Mathworks Inc., Natick, MA). Signals were epoched from − 1000 ms to 1000 ms relative to the TMS pulse. As the data samples where the TMS pulse appears are irretrievable, they were deleted (from − 1 ms to 10 ms related to TMS-pulse onset) and cubic interpolated [35]. To remove artifacts present in the signals, which encompassed TMS-induced, muscle, ocular, auditory, and noise-related artifacts, independent component analysis (ICA) was applied. The independent components (ICs) that represented the aforementioned artifacts were manually selected by three experts. The criteria to remove the ICs were based on their trial-averaged amplitude, spatial distribution, and activation and time–frequency maps [31, 35, 36]. Subsequently, bad channel interpolation and bad trial rejection were automatically performed. Finally, a baseline correction was applied using an interval of 800 ms before the TMS pulse onset. Data were resampled to 5 kHz and band-pass filtered between 0.5 Hz and 70 Hz.

### TMS–EEG signal processing/LMFP-AUC computation

Artifact-free TMS–EEG data processing was performed in a region of interest (ROI) composed of the channels covering the DLPFC, i.e., Fp1, Af3, Af7, F1, F3, F5, F7, FC1, FC3, and FC5 [37]. To measure the activity induced by the TMS pulse on this ROI, the area under the curve (AUC) of the local mean field power (LMFP; in combination LMFP-AUC) was computed for each subject. First, the LMFP was calculated following the formula below:

$${\text{LMFP}}\left(t\right)= \sqrt{\frac{[{\sum }_{i}^{K}\left({V}_{i}\left(t\right)-{{V}_{{\text{mean}}}(t))}^{2}\right]}{K}},$$

where *K* is the number of channels, *V*_*i*_ (*t*) is the amplitude of the signal in channel *i* at instant *t*, and *V*_mean_ (*t*) is the mean amplitude of all channels of the ROI at instant *t*. Finally, the AUC was computed by integrating the LMFP signal from 30 to 250 ms after the TMS pulse.

The LMFP-AUC is a widely used neurophysiological measure that represents activity induced by TMS pulses across a specific subset of electrodes of interest [20]. Therefore, it might be interpreted as an index of the cortical reactivity of the area covered by those electrodes. Sham stimulation signals were pre-processed and processed analogously to the active stimulation signals.

### Statistical analysis

Demographic characteristics were compared between healthy controls and patients using independent samples *t* test or Chi-square test wherever appropriate. Similarly, RMT was compared using independent samples *t* test to ensure that stimulation intensities did not differ between groups. Since SE modulation included many different potentially collinear variables, it was reduced to principal components using PCA, following our previous studies [16]. The number of factors retained was determined by scree plot examination. To obtain a stable solution, the PCA was carried out on a larger sample (*n* = 440) containing the participants of this study and reducing the number of electrodes to 32. The auditory oddball task was performed under the same conditions in that sample. Independent *t* tests were performed to compare SE modulation and LMFP-AUC values between groups and to compare active and sham stimulation signals.

The main hypothesis of the study was tested using Pearson correlation analyses between LMFP-AUC and SE modulation values, including patients and HC, and then repeating this analysis separately for each group. Finally, to rule out a major effect of treatment, correlation coefficients between LMFP-AUC and medication dose (based on chlorpromazine equivalents) were also calculated. Data analyses were performed using SPSS statistical software, version 23 for Windows (IBM).

## Results

### Demographic and clinical characteristics

Demographic and clinical characteristics are presented in Table 1. There were no significant differences between patients and HC in age, sex distribution, or educational level.

**Table 1:** Demographic and clinical characteristics

	Healthy controls	Patients	Test statistic	*p* value
Sample size, no	27	22	NA	NA
Age, years	27.56 (11.27)	33.77 (12.25)	*t* = 1.85	0.071
Sex, M/F	14/13	12/10	*χ*^*2*^ = 0.184 (1)	0.668
Education level, years	15.04 (2.07)	13.50 (3.08)	*t* = − 2.00	0.053
Illness duration, months	NA	74.57 (134.57)	NA	NA
Lifetime hospitalizations	NA	1.48 (1.03)	NA	NA
CPZ equivalents	NA	336.48 (184.31)	NA	NA
PANSS positive	NA	13.55 (5.23)	NA	NA
PANSS negative	NA	14.70 (6.16)	NA	NA
PANSS total	NA	52.05 (19.05)	NA	NA
BNSS total	NA	23.35 (18.98)	NA	NA

Data are given as mean (standard deviation)

### Spectral entropy modulation

**Fig. 1:**
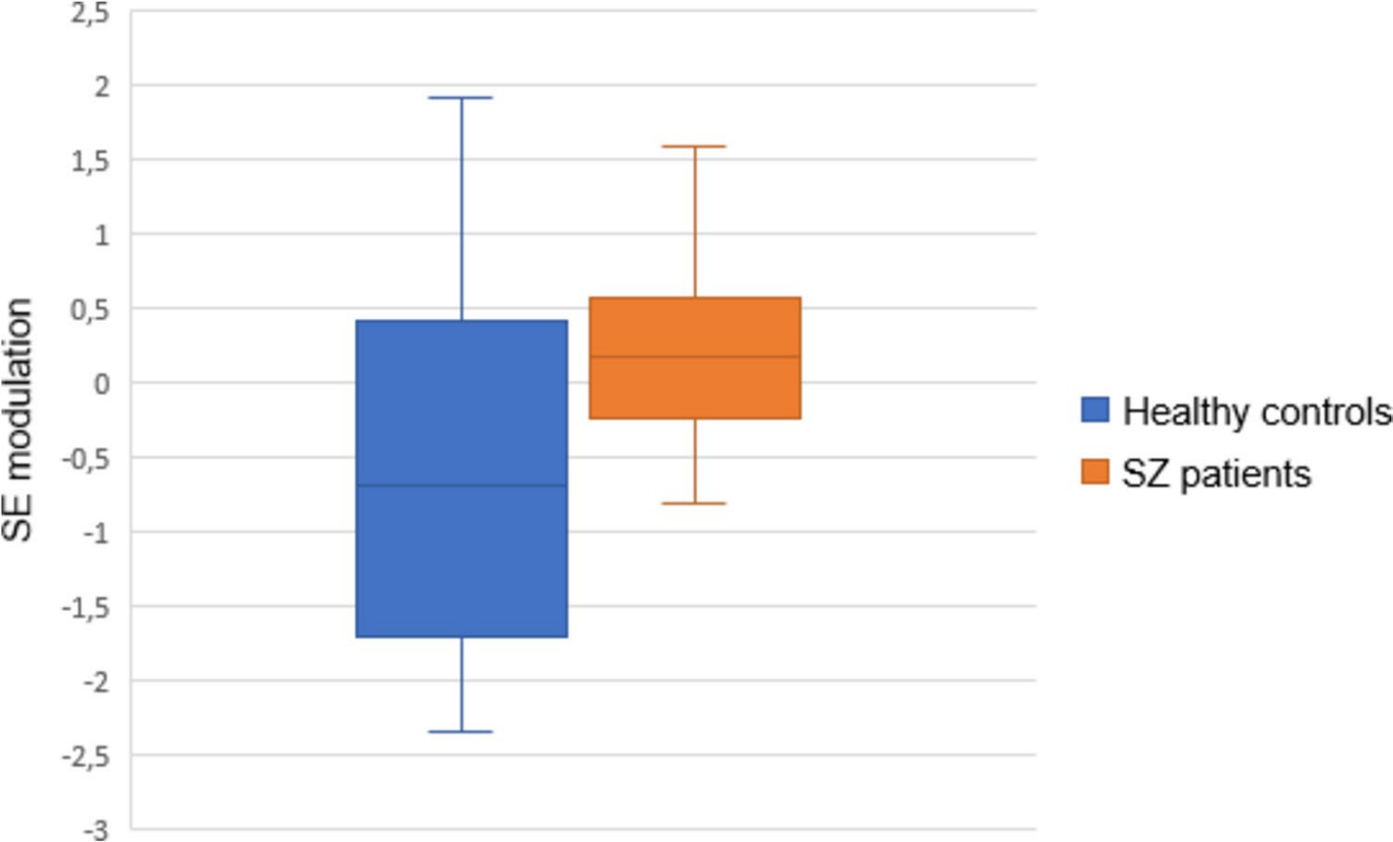
First principal component mean values of SE modulation for healthy controls and schizophrenia patients. Higher mean factor scores represent lower SE modulation

**Fig. 2:**
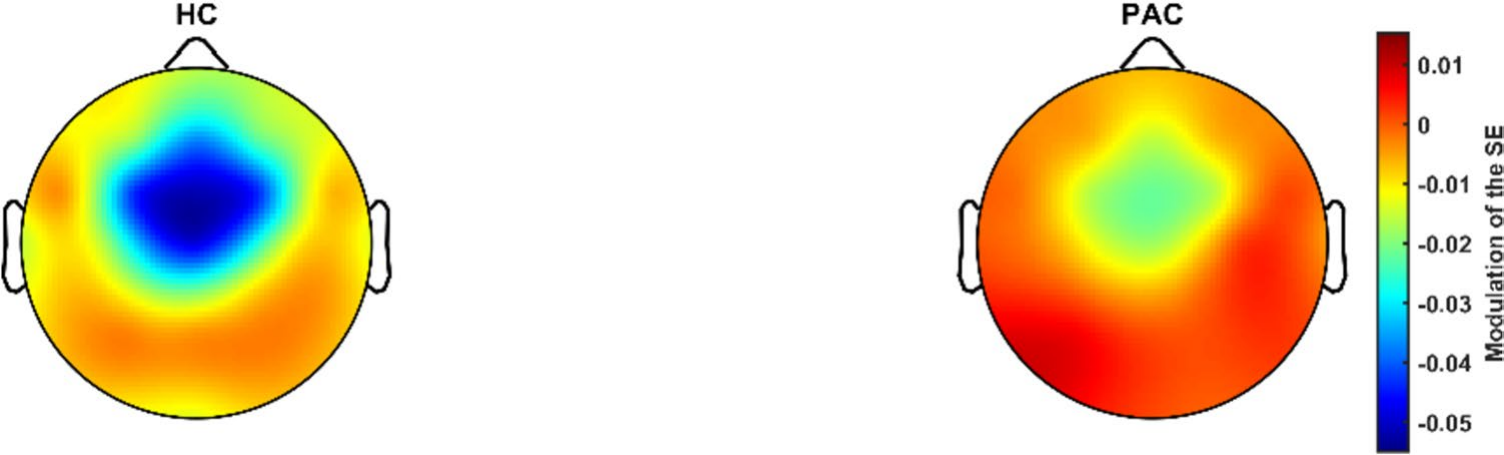
Spectral entropy modulation in healthy controls (left) and schizophrenia patients (right)

In line with our previous studies, the first principal component for SE modulation summarized most of the variance, accounting for 53.94% of the variance (eigenvalue 15.64). All sensors contributed positively to this factor. Thus, higher factor scores represent lower decrease in SE from pre-stimulus to response windows, i.e., lower modulation. Patients showed significantly lower SE modulation than HC (Table 2, Figs. 1 and 2).

**Table 2:** EEG and TMS–EEG characteristics

	Healthy controls	Patients	Test statistic	*p* value
SE modulation	-0.56 (1.19)	0.27 (0.82)	*t* = 2.87	0.006*
RMT	65.11 (9.04)	64.09 (7.46)	*t* = -0.43	0.673
LMFP-AUC	1754.33 (1323.35)	3032.11 (1646.20)	*t* = 3.01	0.004*
LMFP-AUC sham	367.70 (80.18)	363.93 (95.15)	*t* = -0.136	0.893

SE modulation expressed as first principal component values

*RMT*, resting motor threshold

*p < 0.01

### Resting motor threshold

There were no significant differences between patients and HC in resting motor threshold mean values (Table 2).

### LMFP-AUC active stimulation

**Fig. 3:**
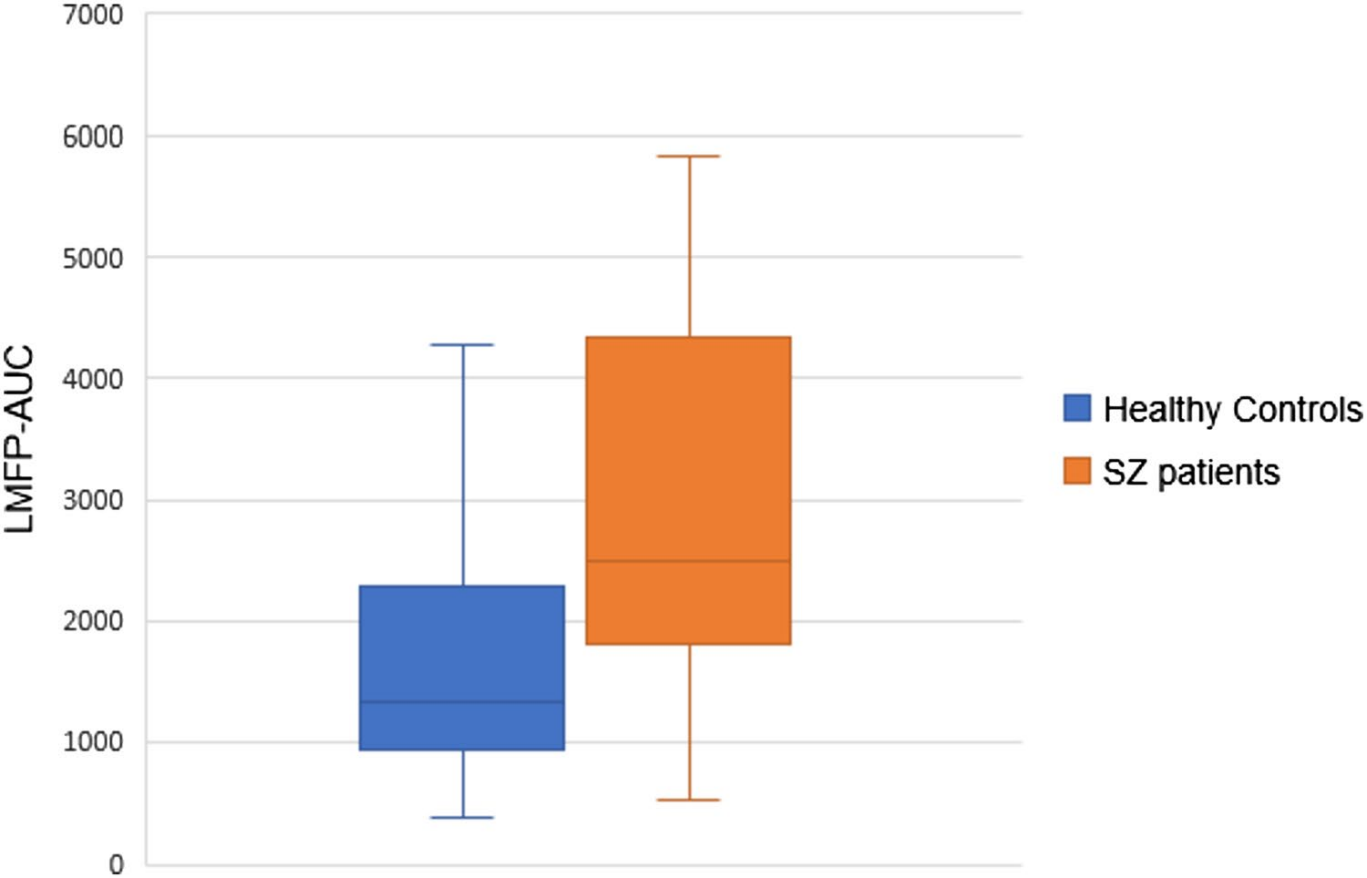
LMFP-AUC mean values for active stimulation for healthy controls and schizophrenia patients

**Fig. 4:**
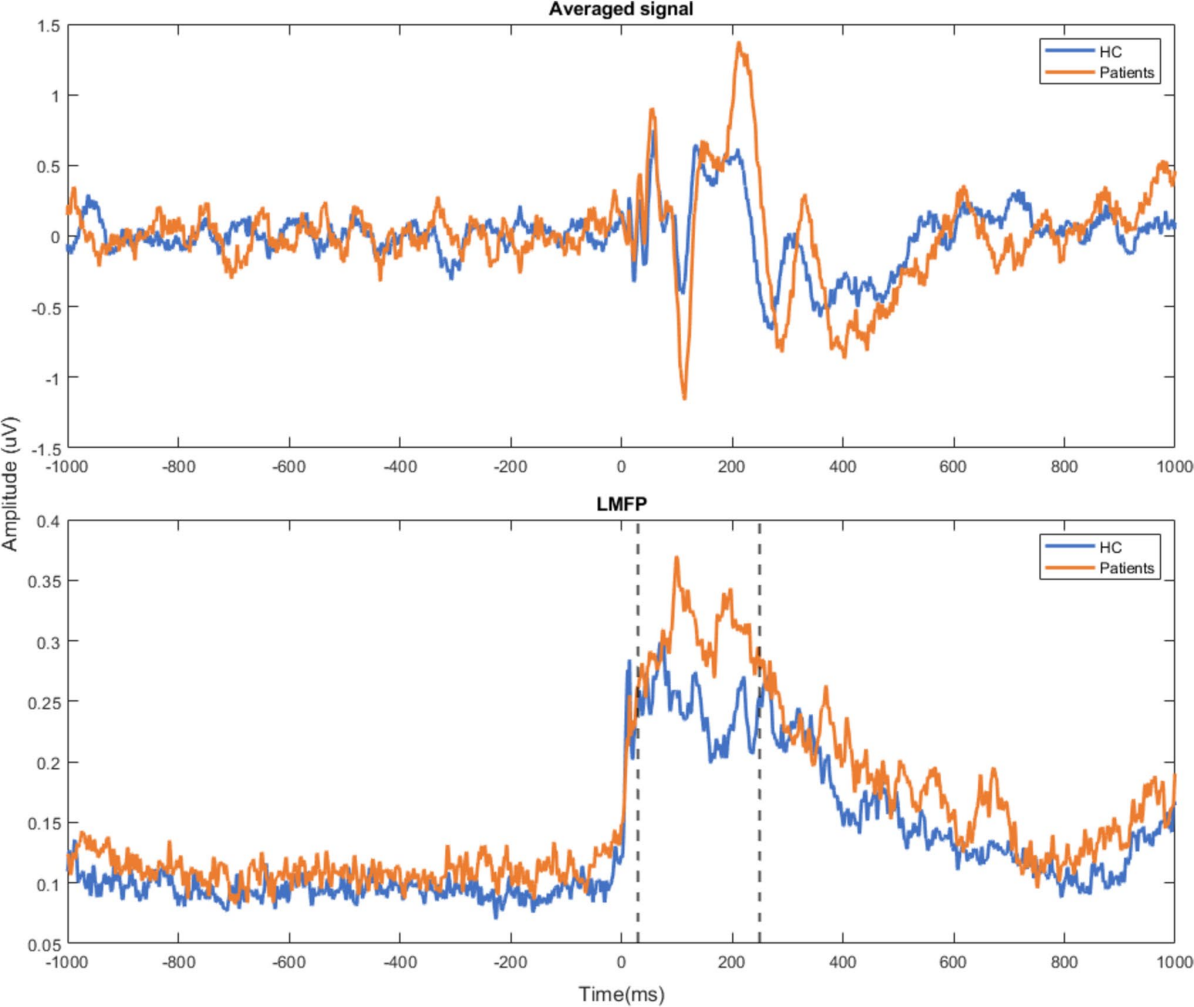
Averaged EEG signal of DLPFC channels (upper panel) and LMFP values (lower panel) after receiving active TMS stimulation for patients (orange line) and healthy controls (blue line)

Schizophrenia patients showed significantly higher LMFP-AUC for active stimulation than healthy controls (Table 2, Figs. 3 and 4). To rule out a potential pre-TMS excitability effect on these results, we decided to compute the LMFP-AUC pre-TMS, specifically between – 230 and – 10 ms relative to the TMS-pulse onset (i.e., so that the time window has the same length as the one used for the LMFP-AUC calculation) for each subject. On average, schizophrenia patients do have a greater LMFP-AUC value compared to healthy controls in this pre-TMS window. However, no statistically significant differences were found between both groups (*p* value of a Wilcoxon text = 0.231).

### Differences between active and sham stimulation

Active stimulation resulted in significantly higher LMFP-AUC compared with sham stimulation when considering the whole sample (*t* = 10.21, *p* < 0.001), and schizophrenia patients (*t* = 8.64, *p* < 0.001) and healthy controls (*t* = 6.88, *p* < 0.001) alone. There were no significant differences between patients and HC in LMFP-AUC sham mean values (Table 2).

### Association between LMFP-AUC and SE modulation

**Fig. 5:**
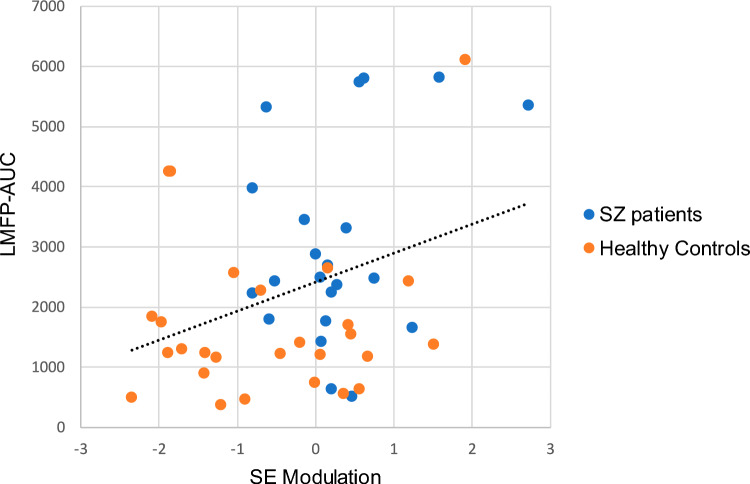
Association between the first principal component mean values of SE modulation and LMFP-AUC mean values and the corresponding best-fitting line considering the whole sample of the study

LMFP-AUC was significantly related to SE modulation (*r* = 0.336, *p* = 0.018) when considering both HC and patients (Fig. 5). This relationship was not significant when considering patients alone (*r* = 0.321, *p* = 0.145) or HC alone (*r* = 0.164, *p* = 0.412).

### Association between LMFP-AUC and medication dose

There was no significant correlation between LMFP-AUC values and medication dose (*r* = 0.299, *p* = 0.177).

## Discussion

To the best of our knowledge, this is the first study assessing the relationship between EEG modulation during a cognitive task and the brain’s excitatory/inhibitory balance in DLPFC evaluated through TMS–EEG in healthy controls and schizophrenia patients. Compared to healthy controls, patients showed a deficit in EEG activity modulation during a cognitive task and higher cortical reactivity following TMS single pulses. Our key finding implies that SE modulation is associated with the amplitude of the evoked response to TMS single pulses in the left DLPFC.

In line with previous studies [12–14], our results replicate a deficit in EEG activity modulation during a cognitive task in schizophrenia patients. This deficit has been shown to be unrelated to psychopharmacological treatment [14] and might reflect a deficit in the synchronization of neural assemblies that underlie cognitive activity. Moreover, patients showed higher cortical reactivity following TMS single pulses in the DLPFC compared to healthy controls. A possible neurophysiological underpinning of these findings may be related to the decreased inhibitory function previously described in schizophrenia [8, 26, 27] that could lead to a baseline cortical hypersynchrony. Inhibitory alterations are also coherent with other replicated alterations in schizophrenia patients, such as P50 gating deficits [38, 39]. Specifically, GABA_B_ receptor-mediated cortical inhibition is thought to underlie sensory gating [40] and it has been also shown to be altered in schizophrenia patients in TMS studies using long interval cortical inhibition [41] and cortical silent period [42].

Our data revealed a positive relationship between SE modulation and the amplitude of the evoked response in the left DLPFC to TMS single pulses. In other words, the higher amplitude of the evoked response to TMS stimulation was related to a decreased task-related modulatory capacity of the EEG. This association agrees with previous studies showing that higher baseline connectivity strength [15, 16] and theta power [17] are similarly associated with a decrease in the modulatory capacity of EEG during the same cognitive task as the one used in our study. These findings altogether could indicate that a larger excitatory activity at baseline is related to a decreased task-related modulatory capacity of the EEG.

It is worth noting that the association between SE modulation and LMFP-AUC after TMS stimulation was only statistically significant when considering the entire sample. This may suggest the idea that this relation would be better considered dimensional rather than categorical. In this way, the excitatory/inhibitory imbalance may only apply to some subtypes within the schizophrenia syndrome, where LMFP-AUC was clearly increased, and correspondingly with basal hypersynchrony and decreased modulatory capacity of the EEG. Accordingly, we have recently reported the existence of a biotype within psychosis primarily characterized by a large cognitive deficit and specific neurobiological alterations, including increased baseline connectivity strength values [43]. This specific alteration was not shared with the other identified biotype which included more preserved patients. Consistently, one recent study reported a biotype with higher cognitive control deficits and associated with overactive neural responses, not present in the identified preserved cognitive biotypes [44]. We did not find statistically significant correlations between LMFP-AUC and SE modulation when considering patients alone, although correlation coefficients in this group were similar to those of the whole sample. This suggest the interest of increasing the sample size and the possibility of finding different biotypes on this basis.

The specificity of the inhibitory dysfunction for schizophrenia may be questioned by studies showing higher LMFP-AUC following TMS single pulses over DLPFC in other clinical populations, such as major depressive disorder (MDD) [45, 46]. Like schizophrenia, MDD may involve, at least in some cases, the dysregulation of cortical inhibitory and excitatory mechanisms [47, 48]. It may be explored whether a common underpinning involving a GABA dysfunction may help characterizing potential biotypes in these syndromes. Nevertheless, to the best of our knowledge, a decreased modulation of EEG activity during a cognitive task and a hypersynchronous basal state has not been shown in MDD, suggesting that the consequences of inhibitory dysfunction may differ in both syndromes.

Some limitations should be considered when interpreting the findings of this study. First, the sample size was relatively small. A larger sample size may reveal significant relationships in the patients studied alone and may help to address differences in inhibitory function in different patients subgroups. Second, we did not use neuronavigation. to localize the left DLPFC. However, in line with previous studies in the field, the coil was placed between the F3 and F5 electrodes, a position that provides the most accurate estimation of the left DLPFC [29–31]. Third, we cannot completely rule out the possible contamination of the TMS-EEG signal by TMS-induced somatosensory and auditory artifacts. However, we analyzed LMFP-AUC as a measure of cortical reactivity instead of looking at isolated potentials, where the potential contamination could have been more problematic. Fourth, this is a correlational study that describes association, but not causation. Finally, our study design cannot fully disentangle the contribution of excitatory vs. inhibitory mechanisms to the EEG activity modulation during a cognitive task. Future studies should include other measurements to try to solve this, such as spectroscopy or paired-pulse paradigms.

In conclusion, our study successfully replicated the fact that, compared to healthy controls, schizophrenia patients showed a deficit in EEG activity modulation during a cognitive task. Moreover, it also revealed that patients display higher cortical reactivity following TMS single pulses applied over the left DLPFC. Furthermore, our data highlight a potential relationship between SE modulation during a cognitive task and the amplitude of the evoked response to TMS single pulses in the left DLPFC in both healthy controls and patients. These findings provide novel insight into the neurophysiological underpinnings of potentially different subgroups of schizophrenia patients.

### Supplementary Information

Below is the link to the electronic supplementary material.

Supplementary file1 (DOCX 24 kb)

## Data Availability

The dataset that support the findings of this study is available from the corresponding author upon request.
