# Supplementary material for: Relation between task-related activity modulation and cortical inhibitory function in schizophrenia and healthy controls: a TMS–EEG study
Source: Eur Arch Psychiatry Clin Neurosci. 2024 Jan 19;274(4):837–47. doi: 10.1007/s00406-023-01745-0 (PMC11127880; doi:10.1007/s00406-023-01745-0)
Supplement: Supplementary file 1 — Supplementary file1 (DOCX 24 kb) [file 406_2023_1745_MOESM1_ESM.docx]

**Supplementary material**

**Relation between task-related activity modulation and cortical inhibitory function in schizophrenia and healthy controls: A TMS-EEG study**

Inés Fernández-Linsenbarth^a,1^, Gema Mijancos-Martínez^b,c,1^, Alejandro Bachiller^b,c^, Pablo Núñez^d,e,f^, Víctor Rodríguez-González^e,f^, Rosa M. Beño-Ruiz-de-la-Sierra^a^, Alejandro Roig-Herrero^a,g^, Antonio Arjona-Valladares^a^, Jesús Poza^e,f,h^, Miguel Ángel Mañanas^b,c,f^, Vicente Molina^a,i,j,*^

^a^ Psychiatry Department, School of Medicine, University of Valladolid, Valladolid, Spain

^b^ Biomedical Engineering Research Centre (CREB), Department of Automatic Control (ESAII), Polytechnic University of Catalonia, Barcelona, Spain

^c^ Institute of Research Sant Joan de Déu, Barcelona, Spain

^d^ Coma Science Group, CIGA-Consciousness, University of Liège, Liège, Belgium

^e^ Biomedical Engineering Group, University of Valladolid, Valladolid, Spain

^f^ CIBER of Bioengineering, Biomaterials and Nanomedicine (BICER-BBN), Madrid, Spain

^g^ Imaging Processing Laboratory, University of Valladolid, Valladolid, Spain

^h^ Instituto de Investigación en Matemáticas (IMUCA), University of Valladolid, Valladolid, Spain

^i^ Psychiatry Service, Clinical Hospital of Valladolid, Valladolid, Spain

^j^ Neurosciences Institute of Castilla y Léon (INCYL), University of Salamanca, Salamanca, Spain

^1^ Inés Fernández-Linsenbarth and Gema Mijancos-Martínez contributed equally to the article.

****Corresponding author:*** Vicente Molina. Dept. of Psychiatry, School of Medicine, University of Valladolid. Av. Ramón y Cajal, 7. Valladolid 47005, Spain. Tel: +34 983 423 200. [vicente.molina@uva.es](mailto:vicente.molina@uva.es)

*Spectral entropy (SE) analysis*

The continuous wavelet transform (CWT) is a useful tool that can provide information about the time-varying properties of the ongoing EEG, which makes it well suited for the study of ERPs [1]. The CWT enables flexible control over the time and frequency resolutions, facilitating the detection of dynamic ERP components [2].

The “mother wavelet” has to be selected carefully to enable a fit that is biologically plausible [3]. In the present study, the Morlet wavelet was used for this purpose, with two of its parameters (center frequency and bandwidth) set to 1 to obtain a good balance between time and frequency resolutions [4]. The CWT is defined as the convolution of a signal *x*(*t*) with a scaled and translated version of the “mother wavelet”:

$\mathrm{CWT}\left( k,s \right)=\frac{1}{\sqrt{s}}\cdot\int_{-\infty}^{+\infty} x\left( t \right)\cdot\varphi^{*}\left( \frac{t-k}{s} \right)dt,$ Eq. (S.1)

where *s* is the scale factor, *k* is the time interval and *φ*(*t*) is the “mother wavelet” [5]. The wavelet scalogram (WS_n_) is the normalized square of the absolute value of the CWT and encapsulates the distribution of the energy of the signal as a time-frequency representation. WS_n_ values range from 0 to 1 and is defined as follows:

${WS}_{n}\left( k,s \right)=\frac{\left| CWT(k,s) \right|^{2}}{\sum_{k} \left| CWT(k,s) \right|^{2}}$ Eq. (S.2)

Spectral entropy (SE) is a time-dependent measure derived from Shannon entropy that characterizes the irregularity of the EEG signal. The CWT-derived SE is defined as follows [6]:

$\mathrm{SE}\left( t \right)=-\frac{1}{\log\left( M \right)}\cdot\sum_{f} \mathrm{WS}_{n}\left( k,s \right)\cdot\log\left[ \mathrm{WS}_{n}\left( k,s \right) \right],$ Eq. (S.3)

where *M* is the length of the signal, while *k* and *s* represent the time interval and the scale respectively.

If a signal displays a large range of spectral components, such as white noise, this will lead to high values of SE (closer to 1), while a signal with fewer spectral components will result in smaller values of SE (closer to 0).

In this study, SE was averaged on two windows: i) the pre-stimulus window (300 ms before the stimulus to the stimulus onset) and ii) the response window: (150 ms-450 ms from the onset of the stimulus). SE values were computed for each subject and window, and then averaged across trials (single trial approach [3]). We defined SE modulation as the change in SE from the pre-stimulus to the response window.

**References**

1. Tallon-Baudry C, Bertrand O, Delpuech C, Pernier J (1996) Stimulus specificity of phase-locked and non-phase-locked 40 Hz visual responses in human. J Neurosci 16:4240–4249. https://doi.org/10.1523/jneurosci.16-13-04240.1996

2. Samar VJ, Bopardikar A, Rao R, Swartz K (1999) Wavelet analysis of neuroelectric waveforms: A conceptual tutorial. Brain Lang 66:7–60. https://doi.org/10.1006/brln.1998.2024

3. Roach BJ, Mathalon DH (2008) Event-related EEG time-frequency analysis: An overview of measures and an analysis of early gamma band phase locking in schizophrenia. Schizophr Bull 34:907–926. https://doi.org/10.1093/schbul/sbn093

4. Núñez P, Poza J, Bachiller A, et al (2017) Exploring Non-Stationarity Patterns in Schizophrenia: Neural Reorganization Abnormalities in the Alpha Band - PubMed. J Neural Eng 14

5. Rioul O, Vetterli M (1991) Wavelets and Signal Processing. IEEE Signal Process Mag 8:14–38. https://doi.org/10.1109/79.91217

6. Gomez-Pilar J, Poza J, Bachiller A, et al (2015) Neural Network Reorganization Analysis During an Auditory Oddball Task in Schizophrenia Using Wavelet Entropy. Entropy 17:5241–5256. https://doi.org/10.3390/e17085241
